# Materials fatigue prediction using graph neural networks on microstructure representations

**DOI:** 10.1038/s41598-023-39400-2

**Published:** 2023-08-02

**Authors:** Akhil Thomas, Ali Riza Durmaz, Mehwish Alam, Peter Gumbsch, Harald Sack, Chris Eberl

**Affiliations:** 1 Fraunhofer Institute for Mechanics of Materials, Freiburg, Germany; 2 Chair of Micro and Materials Mechanics, Department of Microsystems, University of Freiburg, Freiburg, Germany; 3 Télécom Paris, Institut Polytechnique de Paris, Paris, France; 4 Institute for Applied Materials-Reliability and Microstructure (IAM-ZM), Karlsruhe Institute of Technology, Karlsruhe, Germany; 5 FIZ Karlsruhe–Leibniz Institute for Information Infrastructure, Karlsruhe, Germany; 6 Institute for Applied Informatics and Formal Description Systems (AIFB), Karlsruhe Institute of Technology, Karlsruhe, Germany

**Keywords:** Characterization and analytical techniques, Design, synthesis and processing

## Abstract

The local prediction of fatigue damage within polycrystals in a high-cycle fatigue setting is a long-lasting and challenging task. It requires identifying grains tending to accumulate plastic deformation under cyclic loading. We address this task by transcribing ferritic steel microtexture and damage maps from experiments into a microstructure graph. Here, grains constitute graph nodes connected by edges whenever grains share a common boundary. Fatigue loading causes some grains to develop slip markings, which can evolve into microcracks and lead to failure. This data set enables applying graph neural network variants on the task of binary grain-wise damage classification. The objective is to identify suitable data representations and models with an appropriate inductive bias to learn the underlying damage formation causes. Here, graph convolutional networks yielded the best performance with a balanced accuracy of 0.72 and a F_1_-score of 0.34, outperforming phenomenological crystal plasticity (+ 68%) and conventional machine learning (+ 17%) models by large margins. Further, we present an interpretability analysis that highlights the grains along with features that are considered important by the graph model for the prediction of fatigue damage initiation, thus demonstrating the potential of such techniques to reveal underlying mechanisms and microstructural driving forces in critical grain ensembles.

## Introduction

The notion of polycrystalline microstructure embodies a network of grains containing a variety of crystallographic defects. This defect network is a consequence of the processing history and governs the majority of the material’s properties. Therefore, the microstructure acts as a central element to establish so-called process-structure-property (PSP) relationships for polycrystalline materials. Numerous PSP chains are of interest to material scientists. However, for many of them, due to pronounced problem complexity or computational constraints, physics-based models fail to capture the underlying mechanisms comprehensively. Depending on the property of concern, for its forward prediction, microstructure representations with distinct levels of detail are required. While, for instance, an estimation of yield strength in a first approximation can be addressed with average grain size, predicting fatigue damage initiation sites necessitates nuanced and comprehensive microstructure representations. Latter is owed to the fact that there is a pronounced microstructure sensitivity in the regime of cyclic microplasticity, i.e. when the loading barely exceeds the materials’ onset of plasticity and irreversibility^1^. In this high cycle fatigue (HCF) scenario, predicting fatigue damage and crack initiation sites in metallic alloys is essential as it dictates the overall fatigue life. However, for this regime, the mechanistic understanding is incomplete, especially where sophisticated technical alloys are concerned. For such high-complexity problems with inchoate domain knowledge, data-driven techniques are promising candidates to find meaningful relations in the data. In particular, machine learning (ML) techniques can be employed, which enjoyed popularity and success to an extent where such models replaced or complemented physics-based models during the past decade. Consequently, a wealth of tailor-made techniques is available for distinct input data representations. This spoils us for choice and raises the question of how to ideally represent a microstructure and which model to utilize for a specific task such as predicting fatigue damage sites.

A straightforward representation is pixel or voxel data for which convolutional neural networks (CNN) often provide appropriate inductive bias. Meanwhile, these models are well established in materials science, especially for tasks such as segmentation and prediction of damage and crystallographic phases^2–6^. However, for predicting microstructural fatigue damage sites, where high-dimensional data is necessary to capture the interactions between various influence factors, pixel/voxel-based approaches are often infeasible due to their pronounced computational demand. Another way to represent microstructure is by clustering pixel/voxel regions, corresponding to physical concepts such as grains, to individual data instances with multidimensional features extracted and aggregated from the regions. This results in a tabular representation that can be utilized in conventional ML approaches such as random forests (RF) and support vector machines (SVM). In literature, grain-level representations in conjunction with conventional ML models have been utilized to predict twinning events^7^ and stress hotspot localization^8^ within polycrystals. Conventional models are faster to train and easier to optimize than CNN due to their fewer training and hyperparameters but require that the input features provided are discriminative to the task at hand. In contrast, in models like multilayer perceptrons (MLP) the requirement for high feature quality is discounted since such models also learn to combine and find useful features from the initial inputs.

An issue with the tabular representation is that dependencies between individual grains are not easy to model since this representation assumes grains to be independent and identically distributed and considers them as isolated data instances. Instead, representing microstructures as a graph can help model various dependencies and interactions between individual grains. This can be done by modeling grains as nodes and dependencies between them as relations in a graph. In comparison to pixel or voxel-based techniques, computational graphs are non-Euclidean in nature and permit a physical and flexible description of microstructures and their entities. Not being restricted to a regular grid implies that multi-scale problems, such as materials fatigue, can be addressed efficiently. Amongst others, this facilitates considering heterogeneous graphs composed of grain and inclusion node entities. Since both entities exhibit different sizes, representing them as pixels/voxels with a single image resolution cause either information loss or computational overhead.

The graph representations of materials microstructures can be utilized by several ML algorithms designed to work on graphs. One class amongst them that can leverage the structure/neighborhood information is the graph neural network (GNN) introduced by Scarselli et al.^9^ From the initial GNN model, numerous variants have been derived which can operate on specific graph types and differ in how the node information is transformed, aggregated across neighbor nodes, and updated. The message-passing neural network (MPNN) framework proposed by Gilmer et al.^10^ unifies several of these GNN variants using a generalized description. The graph convolutional network (GCN) introduced by Kipf et al.^11^ is the generalization of the convolution to the graph domain and operates in the spectral domain. GCNs were originally proposed for semi-supervised node classification problems and are trained in a transductive setting. The graph isomorphism network (GIN) architecture proposed by Xu et al.^12^ was based on two characteristic changes compared to GCN. Instead of using a weighted average to aggregate the states from neighbor nodes, GINs perform a simple summation. Secondly, in GIN, a few MLP layers increase the expressivity when updating the target node representation based on the message aggregated from its neighbor nodes. These changes are designed to improve the sensitivity of the graph embedding. Most of these methods apply to graphs that contain a single node type and typically also a single edge type, i.e., homogeneous graphs.

Another extension to graph neural network techniques attempted to encode structural properties such as molecular bond angles in an additional line graph^13,14^. Similarly, the crystallographic unit cell structure was encoded in graph representations to predict energy band gaps from multi-fidelity ab-initio simulations was proposed previously^15^. Yang et al.^16^ use GNNs to predict atom-level properties, including stress fields and energy distributions, by modeling a few grains along with contained structural defects. Instances, where the microstructure topology was captured in graphs were presented to predict the stored elastic energy functional^17^, grain-scale toughness^18^, effective magnetostriction^19^, and deformation twinning^20^. Graph-based approaches and the notion of message passing between node entities provide an inductive bias that might be suitable to learn aspects such as interactions within grain ensembles. This entails, for instance, concepts such as elastic and plastic incompatibilities or how close damage sites are to each neighboring grain.

**Figure 1:**
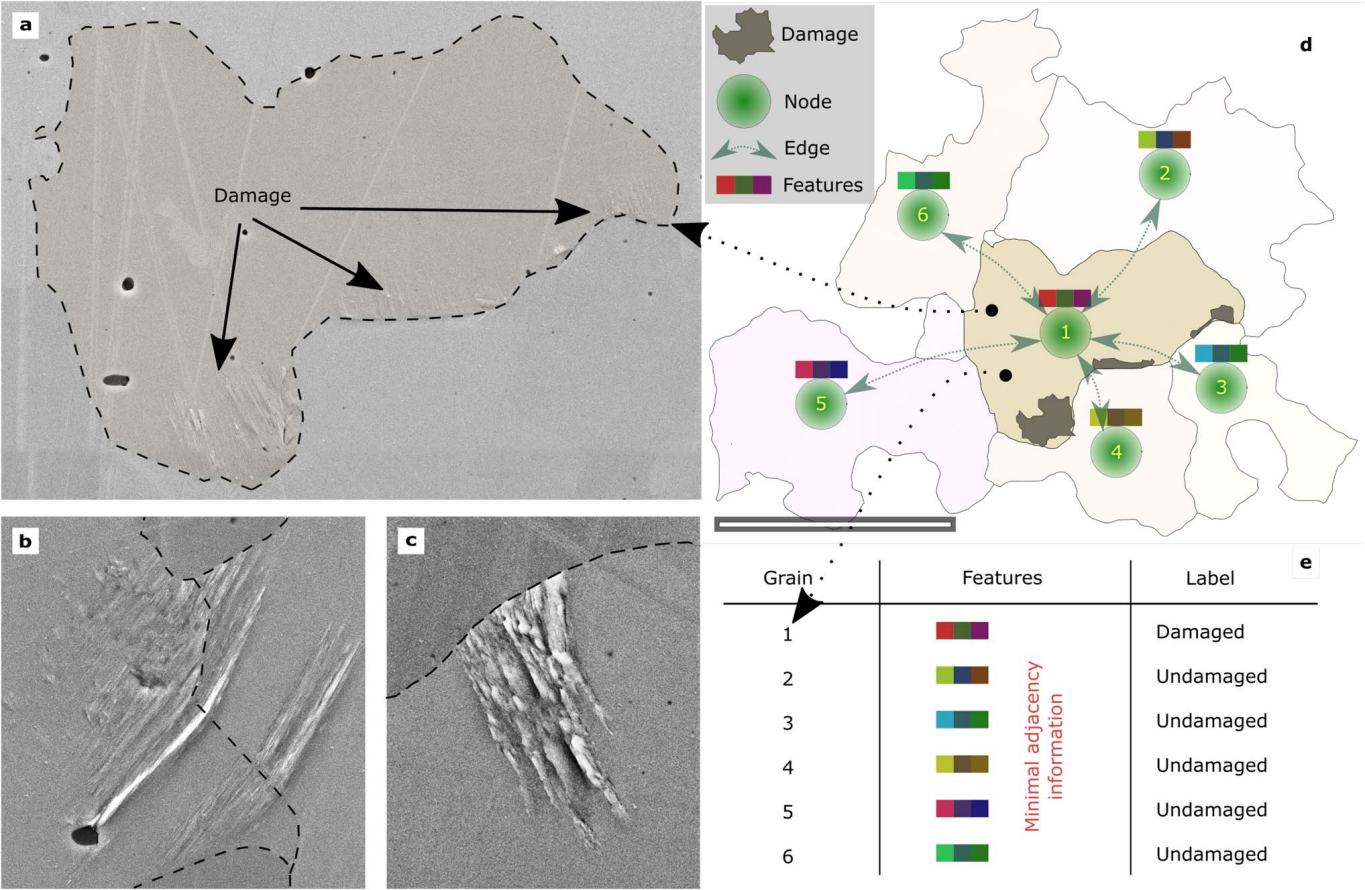
Example protrusion instances and the conversion of a grain neighborhood into graph and tabular representations. The scanning electron microscopy images in subfigures (**a**)–(**c**) display protrusions that are distributed, transition through a grain boundary, and are delimited by a grain boundary, respectively. For the grain in (**a**), subfigure (**d**) illustrates the surrounding microstructure and its graph representation while subfigure (**e**) displays its tabular representation. The full list of features computed for each grain is provided in Table 4. The micron bar in subfigure (**d**) corresponds to 50 μm.

In this work, we utilize multimodal data sets from correlative microscopy^21^ to derive fatigue feature representations and assess their suitability to predict high cycle fatigue damage formation. In particular, we address the formation of slip markings (see Fig. 1a,b,c) that pose rare events and act as precursors for crack initiation. A series of known microstructural aspects affect slip marking formation, including the grains’ size and tendency to accommodate multiple slips, elastic incompatibilities between adjacent grains, the initial dislocation density, the presence of precipitates, and the involved grain boundaries’ resistance to slip transmission to name a few. The infrequency of slip-marking formation not only prolongs the acquisition of statistically representative data sets but also culminates in an inherent data imbalance that needs to be accounted for during learning. Another challenge lies in the time-efficient acquisition of complete feature representations. To date, no individual characterization method captures all influence factors while also complying with testing requirements such as high frequent cycling. As a consequence, the data sets are afflicted by high epistemic uncertainty.

Feature representations that describe the initial specimen state and loading scenario are used to train binary classifiers predicting whether grains will accommodate slip markings. Features utilized entail crystallographic, morphological, and micromechanical descriptors such as Euler angles, grain disorientation angles, grain size, and Schmid factors to name a few. A comprehensive list is provided in Table 4. A significant contribution lies in investigating the efficiency of tabular and graph microstructure representations in conjunction with associated ML methodologies. Figure 1d illustrates the graph representation of the grain and its neighborhood from Fig. 1a. In contrast, Fig. 1e depicts how a tabular representation is built from such grain-wise abstractions. Assessed learning methodologies on the tabular representation comprise balanced random forests (BRF), SVM, and MLP. On the graph representation, the previously mentioned graph neural network approaches GCN and GIN are applied. The shortcomings of different microstructure representations concerning their information loss and their compatibility with learning algorithms are discussed. This includes suitable graph neural network extensions to account for nuanced feature interactions across connected grain nodes.

## Results: modeling grain-wise damage with binary classifiers

This section presents the performance of classifiers trained to discern grains that will contain fatigue slip markings from those grains resilient to surface damage accumulation. As a prerequisite, an unprecedented benchmark data set that will facilitate such a study was prepared and published^22^. The data set originates from the multimodal fatigue experimental data of ferritic steel specimens presented in Durmaz et al.^23^. This data is post-processed to generate grain-level feature descriptors and slip marking annotations, the former acting as inputs and the latter acting as labels for the ML models in a tabular representation. Further, a graph representation of the data set is also generated, where in addition to the grain features, adjacency information is added as edges connecting neighboring grains. The data set has 7633 grains, of which only 311 are annotated with slip markings (making it a highly imbalanced data set, but representative of the HCF loading), with each grain having 120 feature descriptors. Data set generation and feature engineering is further described in "Data set generation" and "Feature engineering" sections.

In the following, results for the individual ML models on both the tabular and graph data sets are compared. For the tabular representation, all conventional ML models shown in Table 1 are trained. However, the graph representation of data requires a separate class of ML models to be trained on them, namely graph neural networks, which are described in "Graph-based machine learning approaches" section. To gauge these results, a comparison is drawn with fatigue indicator parameters (FIP) derived from phenomenological crystal plasticity simulations shown by Durmaz et al.^23^ and summarized in supplementary note 3. This knowledge-based model acts as a baseline model for our classification problem. However, the baseline model results are only available for one specimen side (P2_8 marked) which necessitates the evaluation of ML models only on this data subset for comparison.

We used F_1_-score as the primary metric for evaluation, which penalizes both false positives as well as false negatives, striking a balance between precision and recall metrics and is a popular metric for imbalanced data sets. All utilized evaluation metrics are summarized in the supplementary table 1 and are defined from zero to unity (the higher the better). To study the effect of feature dimensionality reduction, selected ML models were also trained on feature projections made from principal component analysis (PCA). In this case, the node features in the data set were projected onto the first 55 principal components. The number of principal components was chosen such that the components cover >95% of the total variance in the data set.

The hyperparameters optimized for each model and the applied imbalance correction techniques are described in supplementary note 4. We trained all ML models in a transductive setting using the same five-fold data set split. Alternate options for creating folds are elucidated in supplementary note 8. Each model was trained five times with a different random initialization, and the best run as well as the average performance and scatter are documented. The results presented are aggregated from the validation set performance of the respective models from all five folds.

**Table 1:** Performance of machine learning models predicting the formation of protrusions.

Model	Best F_1_-score		Mean F_1_-score	
	Complete data set	P2_8 marked	Complete data set	P2_8 marked
CPFEM Fatemie-Socie	−	0.25	−	−
BRF	0.19	0.20	0.19±0.00	0.20±0.00
BRF-PCA	0.19	0.22	0.19±0.00	0.21±0.01
SVM	0.25	0.25	0.25±0.00	0.25±0.00
MLP	0.29	0.33	0.28±0.00	0.31±0.02
GCN	**0.34**	0.41	0.32±0.01	**0.40**±**0.01**
GCN-BCE	0.32	0.41	0.31±0.01	0.39±0.01
GCN-PCA	**0.34**	**0.44**	**0.33**±**0.01**	**0.40**±**0.03**
GIN	0.33	0.42	0.32±0.01	0.38±0.03

The table compares machine learning approaches operating on the tabular data representation (balanced random forests, support vector machines, and multilayer perceptrons) with those using graph representation directly (graph convolutional network and graph isomorphism network). Both the best and mean performance of each model (from five random initializations) are presented. The left column in each block evaluates the models on the whole data set (aggregating across validation sets of all five folds), and the right column evaluates them only on grains from a single specimen side (named P2_8_marked) for which a CPFEM phenomenological model predicting Fatemi-Socie fatigue indicator parameter is available as a baseline. Some special cases of models were also presented—the GCN and BRF models trained using data transformed by principal component analysis (PCA) and the GCN model trained with binary cross entropy loss without any additional imbalance correction techniques.

The values in bold indicate the best performance achieved per column.

**Table 2:** The confusion matrix of the GCN-PCA model for binary classification on the complete data set.

		Prediction	
		Negative	Positive
Actual	Negative	6844	478
	Positive	154	157

**Table 3:** The confusion matrix of the GCN-PCA model for binary classification on P2_8 marked.

		Prediction	
		Negative	Positive
Actual	Negative	830	72
	Positive	19	30

From Table 1, it can be seen that graph-based models (GCN, GIN, and variants), presented in "Graph-based machine learning approaches" section, perform better than conventional ML models on the complete data set. Compared to MLPs, GCN gives us an improvement of approximately 14% in F_1_-score. A similar observation can be made from the model comparison on a single specimen side. Moreover, in this case, the GCN-PCA model surpasses the baseline performance by 76%. Additionally, it could be seen that using PCA-transformed features results in slight improvements, especially in the GCN case. In the GCN-BCE case, where no measures were taken to account for data imbalance, the performance drops by 0.02–0.03 in F_1_-score as opposed to an imbalance-corrected variant (GCN). During GCN hyperparameter optimization, the amount of GCN layers was altered, and two layers, i.e., taking first and second-order grain neighbors into consideration, proved best. For a detailed assessment of the GCN-PCA model, the confusion matrix evaluated on the full data and the marked side of individual specimen P2_8 is presented in Table 2 and 3, respectively.

A slightly less, but similar, improvement is made by the GIN model as well. In fact, except for the BRF model, all models outperform the baseline. The BRF model learns a decision boundary that culminates in many false positives, as shown in the confusion matrix in Supplementary Table 3. Consequently, the precision metric of the BRF model (see supplementary data) is unsatisfactory and the F_1_-score amounts to 0.19, see Table 1.

It is noteworthy that the results of the graph models differ significantly when individual specimens are considered. This is observable in Table 1 where P2_8 exhibits a well above average F_1_-score. In contrast, the conventional ML models perform similarly on P2_8 and the full data set. This characteristic is addressed in detail in the supplementary note 7.

**Figure 2:**
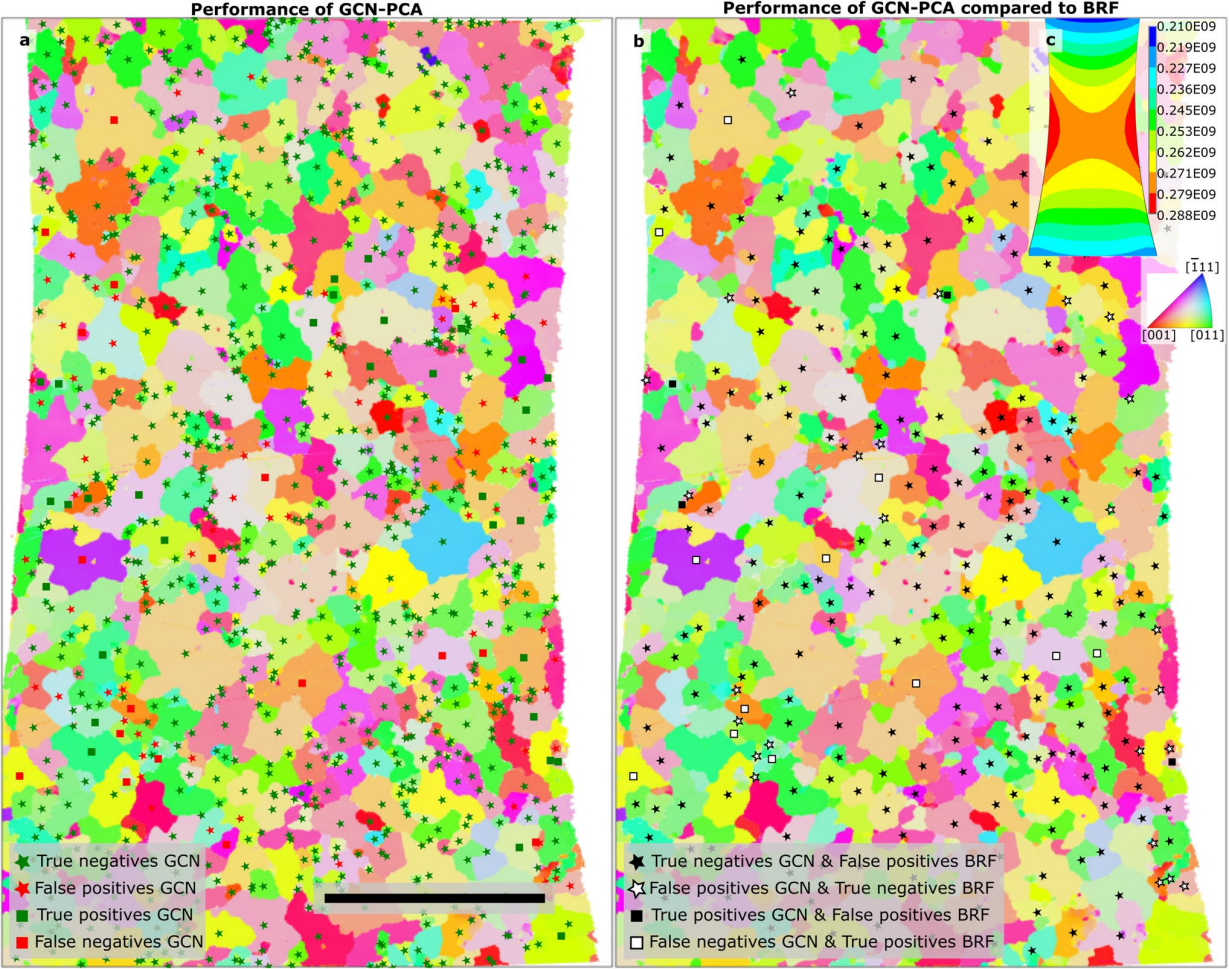
Best model (GCN-PCA) prediction performance visualized and contrasted with the worst model (BRF) performance (see Table 1) by overlaying on inverse pole figure color-coded microstructure maps of P2_8 specimen marked side. Subfigure (**a**) shows the performance of the GCN-PCA. Here, at each grain’s centroid, a symbol is plotted that classifies the model’s prediction in terms of confusion matrix elements. The “square” and “star” symbols indicate damaged and undamaged grains, respectively. A green colored symbol indicates that the model’s prediction for that grain is correct (i.e., either true positive or true negative depending on the symbol), and a red symbol indicates a wrong prediction (i.e., false positives and negatives). Subfigure (**b**) contrasts the prediction performance of GCN-PCA from the BRF model. Here, the symbols denote the same GCN-PCA prediction states as in subfigure (**a**). Solid symbols represent those cases where GCN-PCA predicts correctly and BRF predicts wrong, and vice-versa for the open symbols. For all those grains that do not have a symbol plotted, both GCN-PCA and BRF make the same prediction. The reference direction of the inverse pole figure (IPF) is the specimen normal (ND) [001], and the IPF colormap is inlaid in subfigure (**b**). In subfigure (**c**), the specimen geometry-induced von Mises stress distribution is illustrated. The micron bar in subfigure (**a**) corresponds to 200 $\upmu$m.

For a better assessment of learned model tendencies, the confusion matrix elements encoded as symbols are plotted onto the corresponding grain centroid in the grain map depicted in Fig. 2a. It can be observed that many small grains are correctly classified as undamaged by the GCN-PCA model. However, in the tapered region, there are also small damaged grains identified by the GCN-PCA model as such. Notably, the erroneous predictions are concentrated around the tapered region exposed to high load, cf. Fig. 2a and c. This applies especially to the specimen edge within the tapered region where a high emergence of false positives can be observed. At the edge, the global stress state is more complex and depends strongly on the local geometry which cannot be controlled perfectly during sample fabrication and subsequent polishing. However, the classifier does not predict all grains in this region to be damaged. In contrast, the BRF model predicts all larger grains within the tapered section indiscriminately to contain damage, see Supplementary Figure 1.

In order to analyze the difference between the GCN-PCA and the BRF models, their predictions are compared. Whenever their prediction differs, a symbol is displayed at the grain centroid in Fig. 2b. In this case, solid symbols point to grains where GCN-PCA predicted correctly and BRF predicted wrong, and vice-versa for open symbols. It is notable that the models differ predominantly in the homogeneously loaded region but not immediately at the specimen edge (no symbols are plotted at the left and right of Fig. 2b). Moreover, in regions of low von Mises stress (top and bottom of Fig. 2b), both models agree. The learned decision boundary of the GCN-PCA significantly reduces the false positive predictions as opposed to the BRF model. The GCN-PCA model accounts better for the inherent imbalance of slip marking formation.

## Discussion

Large enterprises in the manufacturing sector contemplate using microstructure-sensitive crystal plasticity simulations and derived fatigue indicator parameter metrics in component design to predict service life and its scatter^24,25^. Provided a crack initiation position, phenomenological CPFE models were shown to predict the paths in which short cracks traverse through the microstructure decently well^25^. However, the hot spots in the mechanical fields or derived FIP metrics typically do not mirror experimentally observed damage or crack initiation sites^23^. To date, such models fail in practice to predict the service life and specifically its scatter in the high-cycle fatigue regime accurately since the microstructural driving forces of crack initiation are not captured comprehensively. In fact, a quantitative understanding of which combination of microstructural features constitutes a potential damage initiation site is largely missing.

In this work, we propose an alternative approach of using statistical GNN models with high expressivity to model microstructures and their plastic response to cyclic loading. Such models provide an adequate inductive bias to learn interactions between adjacent grains and relevant microstructural descriptors. Once such models are well trained, interpretability techniques could be applied to gauge what kind of features the model depends on for making predictions. While CPFEM is limited in domain size, GNN models at inference time can, in principle, produce predictions at the component scale. This permits a thorough analysis of service life scatter, where an engineer is not limited to the simulation of a few statistical volume elements. Moreover, GNN operating on graphs that are non-Euclidean representations implies that multiscale aspects such as grain boundary segregation, pores, and similar can be taken into account more efficiently than in mesh or image-based representations.

The results showed that graph-based models outperform the phenomenological CPFEM baseline and conventional machine learning techniques by large margins when predicting damage locations on this data set. It can be noticed that the absolute value of the F_1_-score, even for the best models, only reaches up to 0.34. However, the F_1_-score does not look at how well the model predicts undamaged grains. The balanced accuracy metric, on the other hand, considers both the labels and the imbalance of the data set. Our best GCN model has a balanced accuracy score of 0.72. Especially considering the complexity of the prediction task, incomplete feature space, and inherent data scarcity, the graph convolutional classifier achieved a very promising performance. Generalizability studies were not conducted due to the limited quantity and the narrow domain (single alloy and heat treatment) of the available data. Techniques such as unsupervised domain adaptation (which removes the need for additional supervisory annotation of data) that performed well on the image-based representation of microstructures^26^ could potentially be utilized also for the graph models once compatible data sets are available. To facilitate better generalization of the GNN models to similar materials and loading conditions, combined GNN-CPFEM frameworks could be designed. In the applied CPFEM modeling, the cause of discrepancy to the experimental data cannot be pinpointed with the available experimental information^23^. Whether the error originates from the microstructure reconstruction, constitutive modeling, FIP damage modeling, or a combination thereof can potentially be identified by conducting feature sensitivity studies on quasi-raw data (crystallographic orientations) and processed features from FIP. Moreover, the applied and other typical CPFE formulations do not incorporate the minuscule subsurface information that is contained within Kikuchi patterns and derived quality metrics of 2D EBSD scans. In contrast to CPFE models, ML approaches provide more flexibility in incorporating such important features without the need for major adjustments in constitutive modeling.

In the GCN-PCA model, the erroneous predictions are often clustered and a large portion is localized to the specimen edge in the tapered region where, assuming a rectangular specimen cross-section, the highest macroscopic stresses occur (cf. Fig. 2a and the stress distribution inlay Fig. 2c). These false predictions by the model can be ascribed primarily to the stress state as well as damage accumulation mechanisms active at these regions being distinct due to a number of potential reasons, including specimen edge rounding, sidewall topography, and residual heat-affected zone from laser-cutting. Additionally, much of that information is not captured in the data set, and on top of it, edge grains are underrepresented in the data which makes it difficult for the model to learn.

The graph models improve upon the conventional ML models in the homogeneously and highly loaded region, cf. Fig. 2b and the orange band in the inlay Fig. 2c. Within this region, the macroscopic stress state is not significantly altered such that microstructural aspects dictate whether damage occurs or not. This indicates that the graph-based approaches manage to learn more second-order relations and nuanced correlations from the data.

**Figure 3:**
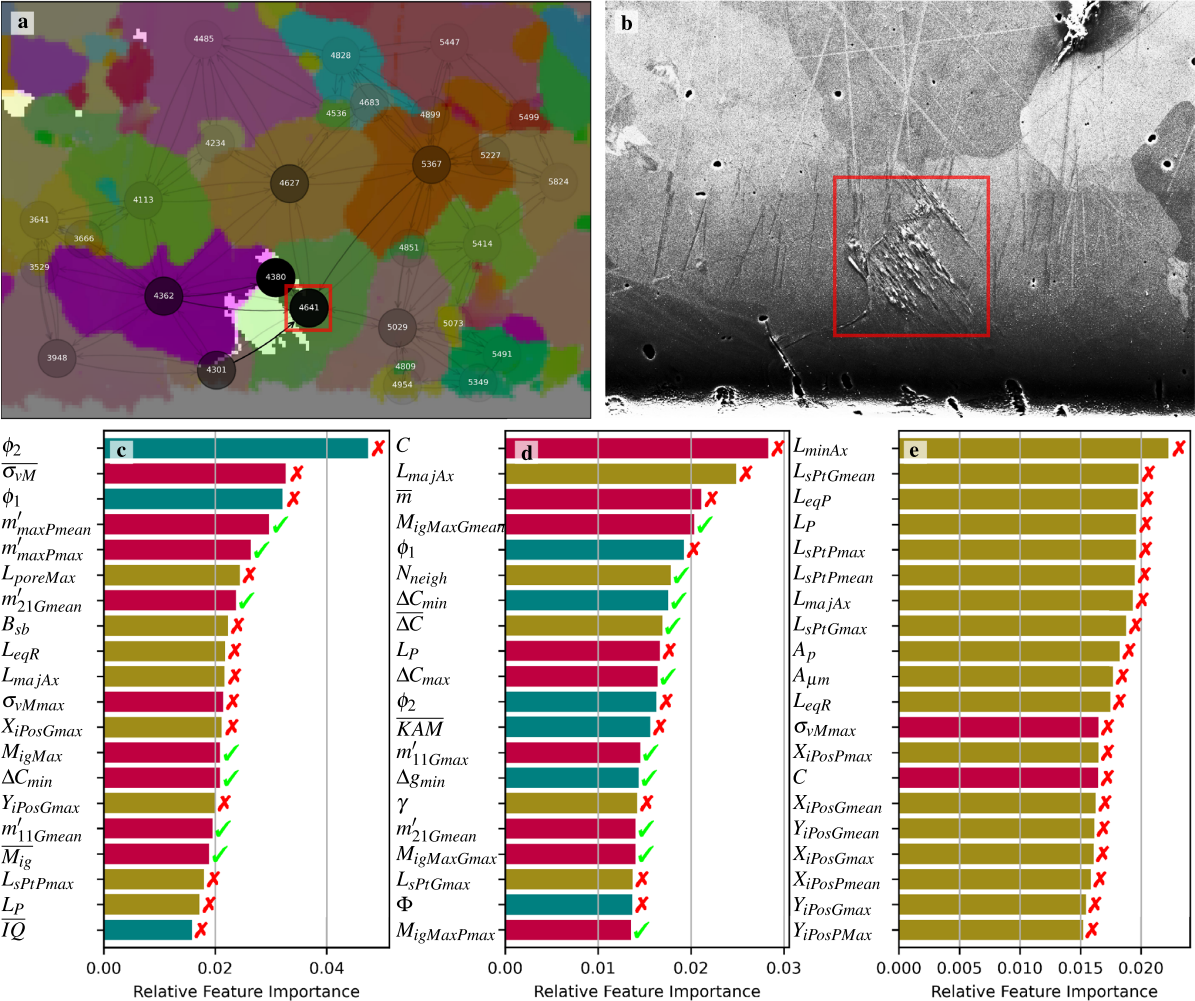
Images showing interpretability analysis results of the GCN model from Table 1 using integrated gradients (IG) attribution (**a**)–(**d**) and the BRF model using the mean decrease in impurity (MDI) metric (**e**). Subfigure (**a**) shows a damaged target grain (#4641) predicted correctly by the GCN model. The microstructure graph and damage (white) are overlayed on the IPF-colored microstructure image. The more opaque the nodes and the edges are, the higher their IG attribution value and thus the higher their importance for the prediction outcome. To complement this, subfigure (**b**) shows the secondary electron SEM image of the damage instance. Subfigure (**c**) shows the importance of specific features for making the particular prediction in subfigure (**a**). In contrast, in (**d**) the joint importance aggregated over all true positive instances in the data set is displayed. The color code and feature symbols for the bar plots in subfigures (**c**)–(**e**) refers to the feature taxonomy introduced in Table 4. The color code in (**c**)–(**e**) refers to the type of the feature: gold refers to “morphological and topological features”, cyan refers to “crystallographic orientation, misorientation and quality-related features”, and red refers to “micromechanical and loading-related features. Similarly, the cross and check marks are transferred from Table 4 to indicate features that utilize information on adjacent grains.

The learned relations can be further explored by determining how the model makes its prediction. For graph models, this can be addressed with interpretability techniques such as integrated gradients (IG) attribution, as introduced in "Interpretability of machine learning models" section. The IG attribution computes an attribution value for the input graph (defined by feature matrix *X* and adjacency matrix *A*) by making interpolations of the graph from a null graph and accumulating gradients from each of those interpolations for each element of *X* and *A* relative to a prediction. IG attributions satisfy several axioms that are considered desirable for an interpretability technique, including the completeness axiom which states that sum of IG value of input features add up to the model’s prediction score for that input^27^. Figure 3 shows an example of IG attribution of the GCN model from Table 1 and compares it with the mean decrease in impurity (MDI) feature importance of the BRF model. The IG attribution for a grain node shows us relatively how important the features of that specific grain are for predicting damage to the target grain. The relative importance of an edge for the target node’s prediction can be observed from the IG attribution of an edge. Relative feature importance charts were made from the IG attributions by aggregating them for a particular feature across all nodes (column-wise sum of *X*). In Fig. 3c,d, such charts for features were calculated from IG attributions relative to either a single grain’s prediction or accumulating from all true positive predictions in the data set. As an ML baseline comparison, the MDI feature importance for the BRF model is computed by looking at how much each feature contributes to reducing the impurity of nodes across all trees in the BRF model ("Interpretability of machine learning models" section). The features computed for each grain node are summarized in Table 4.

**Table 4:** Engineered features containing information on morphology, crystallography, and loading extracted from the multimodal data.

Feature symbol	Description	AG
Morphological and topological features		
$A_{p}$, $A_{\mu m}$	Grain area in number of pixel and physical units (μm^2^)	
$L_{eqR}, L_{eqP}$	Radius, perimeter, of equivalent area circle fitted to the target grain	
$A_{maxNeigh}$, $\overline{A_{neigh}}$	Max. and mean pixel area of adjoining grains	
$N_{neigh}$	Number of neighbor grains	
$L_P$	Perimeter of grain boundary (inner boundaries are neglected)	
*AR*	Aspect ratio of an equivalent area ellipse fitted to the grain	
$L_{majAx}$	Major axis length of an equivalent area ellipse fitted to the grain	
$L_{minAx}$	Minor axis length equivalent area ellipse fitted to the grain	
$\gamma$	Orientation angle of an equivalent area ellipse fitted to the grain	
$B_{sb}, B_{imEdge}$	Booleans indicating specimen boundary grains and grains at edges of the image respectively	
$N_{pore}$	Number of surface pores in grain	
$L_{poreSum/poreMax}$	Accumulated pore diameter and maximum pore diameter within a grain	
$L_{sPt*}$	Max. line intersection grain size in direction of maximally-loaded slip plane trace	
$X_{cen}, Y_{cen}$	Centroid X and Y positions of the grain	
$X_{iPos*}, Y_{iPos*}$	Positions of the intersection between the GB and slip plane trace	
$t_{specimen}$	Thickness of the specimen	
Crystallographic orientation, misorientation and quality-related features		
$\phi _{1}$, $\Phi$, $\phi _{2}$	Mean Bunge Euler angles	
$\Delta g_{min/max}, \overline{\Delta g}$	Min, max, and mean intergranular disorientation angle to any neighbor grain	
*GOS*	Intragranular angular disorientation spread from average orientation, see supplementary equation 6	
${\overline{KAM}}$	Grain-averaged kernel average misorientation, see supplementary equation 5	
$R_{tilt}$	Proportion of tilt boundary candidates ($< 15^{\circ }$ angular deviation between GB segment trace and 2D GB crystallographic misorientation axis) with respect to overall grain boundary length	
$R_{twin}$	Proportion of $\Sigma$3 twin GB trace segments with respect to overall grain boundary length	
${\overline{CI}}$	Grain-averaged EBSD confidence index^42^	
${\overline{IQ}}$	Grain-averaged EBSD image quality^43^	
Micromechanical and loading-related features		
*C*	Stiffness of a grain in the specimen axis direction	
$\Delta C_{min/max}$, $\overline{\Delta C}$	Min, max, and mean of specimen axis direction stiffness difference between adjacent grains	
$\sigma _{vMmax}$, $\overline{\sigma _{vM}}$	Grain mean and max continuum von Mises stress (FEM)	
$m_{max*}$	Grain max of Schmid factors assuming axial tensile load	
$m'_{max/mean*}$	Slip transmission factor considering alignment of slip plane normal and slip direction^44^	
$m'_{ij*}$	Slip transmission factor analogous to $m'$ but considering the crystallographic orientations at the GB of the position ($X_{iPos}, Y_{iPos}$)	
$M_{igMax/Min*}, \overline{M_{ig*}}$	Intergranular misorientation crack factor^30^	
$f_{res}, \sigma _{amp}$	Specimen initial resonant frequency and load amplitude applied during bending test	

The adjacent grain (AG) column, specifically the green check marks, indicates the features which contain information about neighbor grains. All features marked with an asterisk are computed in different ways, using either the grain-mean crystallographic orientations or a statistical measure of the orientation distribution within the target grain (i.e., considering the EBSD orientation samples contributing to the grain individually).

In Fig. 3a, the grains in the direct vicinity of the damage instance are deemed very important by the model (high opacity). The same applies to the edges which connect the grains involved in damage evolution. This indicates that the model learns the patterns in the features that are responsible for damage evolution. Also, the lower involvement of distant grains, e.g., grain ’5447’ or ’4485’, is in accordance with the understanding of ranges of elastic and plastic fields. When assessing the features that the model considers important for this particular prediction, see Fig. 3c, the crystallographic orientation ($\phi _2$, $\phi _1$), $\overline{\sigma _{vM}}$, slip transmission factors ($m'_*$), the grain size ($L_*$), and $B_{sb}$ rank among the most important ones. This is plausible as orientations dictate the propensity for dislocation slip, the stress is elevated in the specimen boundary region, slip transmission affects whether the slip bands and slip markings form at grain boundaries, and the grain size affects dislocation interactions and mobility. The disorientation of neighboring grains affects the elastic misfit resulting in a specific local stress state and the barrier effect of the grain boundary. These two are mechanistically not at all related and affect the damage formation very differently. This motivates introducing features dedicated to specific mechanisms such as slip transmission factors. The boolean designating specimen boundary grains $B_{sb}$ ranking among the most important features implies that the model learns to treat edge grains and other surface grains differently. The aggregated feature importances for all positive instances in Fig. 3d show a similar trend. Additionally, the minimum and maximum intergranular disorientation ($\Delta g_*$) as well as intragranular misorientations represented through *KAM* are considered important. These features determine the effective grain size and contain information on the geometrically-necessary dislocation density. Note that the von Mises stress does not occur as a discriminative feature for the true positive instances. Presumably, this can be traced back to the damage instances and consequentially true positives being located mostly in the high-loaded regions, hence exhibiting small $\sigma _{vM}$ variance.

In the BRF case, see Fig. 3e, it is evident that almost only grain size-related features show up as important features for discriminating damaged and undamaged grain instances. This is in line with the prediction map in Supplementary Figure 1. Seemingly, the features that the classifier learned to depend on do not capture the cause for some small grains developing protrusions. These features are rather one-sided and not as well-balanced across many supposedly relevant descriptors as in the case of the GCN model. Aside from grain morphology features, only the directional stiffness *C* and $\sigma _{vMmax}$ show up, while grain boundary descriptors are completely absent. However, interactions between microtextural and grain boundary descriptors are known to play an important role in an HCF setting^28^. Aside from the missing contextual information in the case of tabular representations, the crystallographic aspects not being learned can be partly ascribed to the fact that during the construction of the individual trees of the BRF bagging classifier, the feature grouping (e.g., Euler angles) was not respected in the feature subsets. Despite a few of the damage instances being localized to pores, pore morphology-related features are underrepresented. This can probably be ascribed to the neglect of the exact pore location. Instead, pore features are extracted for the whole grain while protrusions only cover small portions of grains. Only in Fig. 3c, the maximum pore diameter within the grain ranks among the important features.

**Figure 4:**
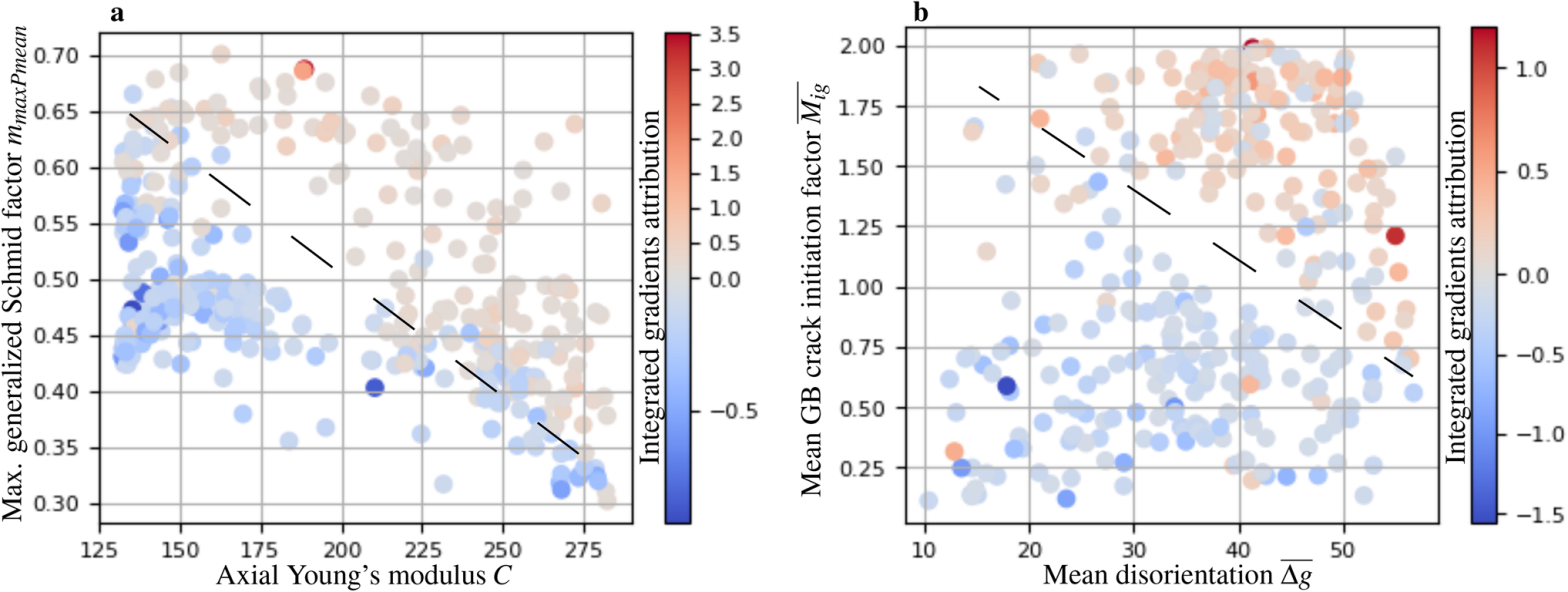
Images showing how different features affect interpretability prediction of damage by an ML model on an average. The plots are calculated for the GCN model from Table 1 using integrated gradients (IG) attribution. Subfigure (**a**) shows the importance of a pair of features (max generalized Schmid factor in the grain ($m_{maxPmax}$) and Young’s modulus of the grain in the axial direction (*C*)) plotted over their value. Each point denotes a grain with the corresponding feature values. The color of the point is calculated as the sum of the average IG attribution value for the specific features across all true positive instances. Subfigure (**b**) shows the respective values for the feature pair mean GB crack factor ($\overline{M_{ig}}$) and mean disorientation of the grain across neighbor grains ($\overline{\Delta g}$).

Figure 3c,d shows some of the features that are deemed important by the best GCN model for making good predictions. But it doesn’t tell us how a feature’s value influences the model’s prediction—whether it attenuates or enhances prediction as damaged. To analyze how the feature values affect the prediction, we create dependence plots, where we can compare the feature value of a particular grain with the IG value attributed to that particular feature in the grain. Specifically, we display a pair of features and color code it with the sum of their IG values (Fig. 4), which would give us the marginal importance of the feature pairs (since IG attributions satisfy the completeness axiom, as described in "Interpretability of machine learning models" section). Dashed lines in the figure show imagined linear decision boundaries separating points that support (IG value > 0) or oppose (IG value < 0) prediction as damage (on an average).

Figure 4 shows dependence plots for two pairs of features that are deemed important by the earlier interpretability analysis. Higher values of Schmid factor mean higher critically resolved shear stress on a slip system, which promotes dislocation activity and slip marking formation. This tendency of higher values of Schmid factor encouraging damage initiation prediction of the grain and its neighborhood is clearly visible in Fig. 4a. Due to the strain-controlled nature of the fatigue experiments we conducted, those grains with high stiffness in the specimen axis direction are exposed to higher stresses. Such grains are more likely to be damaged and this tendency can also be observed in the subfigure a, where grains with high stiffness can be seen promoting damage prediction even when exhibiting smaller max. Schmid factors. Damage initiation in grains with higher stiffness also depends on the stiffness of grains in its neighborhood—stiffer grains surrounded by compliant grains carry more load and are more likely to initiate damage^29^. Such patterns could also be analyzed using GNNs and such interpretability techniques but is considered out of scope of the presented work. Further, in Figure 4b we can see that higher values of GB crack initiation factor^30^ and mean disorientation promote the prediction of damage. While the former was originally proposed as an intergranular crack initiation criterion, it also holds information on the plastic incompatibility between grains and local grain boundary orientation. Thus, the formulation of $\overline{M_{ig}}$ can be interpreted to contain unique information on dislocation impingement on the grain boundary. Considering the irregular grain shapes and the localization of slip markings to specific grain boundary segments (see Fig. 1b,c), further justify the importance of $\overline{M_{ig}}$.

An important aspect to consider when selecting GNN variants is whether they can model relevant interactions between features of neighboring nodes, which is determined mainly by the message passing and aggregation phases ("Graph-based machine learning approaches" section). Such interactions can be very useful for predicting damage from microtexture, for instance, the crystallographic orientations of two adjacent grains determine their compatibility in terms of slip transmission. However, the message passing phase of both GCN and GIN (see Table 5) aggregates features from neighbors before computing any feature combinations, thus ignoring the possible interactions between features of grain pairs. In literature, different approaches to learning sophisticated feature interaction across adjacent nodes have been proposed^31–33^, which might be interesting to model microstructural deformation. The GCN performing slightly better than the GIN, see Table 1 might also be linked to the aggregation of messages across neighbor grains. The GIN model assumes a simple summation of all involved neighbor’s hidden state representations. In the case of low or high node degrees, this form of aggregation can cause vanishing or exploding gradient problems and, therefore, unstable training. This is especially problematic in microstructure graphs like ours with high grain size and morphology variance, where there is a large range of node degrees, i.e., where some grains have only a single neighbor while others have twelve. At the same time, we consider the weighted average summation in GCNs (see Table 5) as non-ideal because it artificially increases the importance of low-degree nodes, typically corresponding to smaller grains with fewer neighbors. Such an aggregation scheme could also potentially make similar embeddings for neighbor grains, which could be the reason why we observe clusters of errors in Fig. 2. GNN literature shows the merits of using multiple aggregators over single aggregators, and scalars to attenuate or enhance messages^34^, which looks like a promising alternative. Graph attention networks (GATs) could also be an interesting choice which is inspired by the usage of attention mechanisms in computer vision and natural language processing^35^. These models perform a weighted sum of messages from neighbor nodes in their aggregation step, where these weights, in contrast to GCN, can be learned, i.e., different neighbor nodes can have different importance when aggregating messages from them. GNN variants with more adequate inductive bias and information aggregation for microstructure graphs will be the subject of future investigations. However, as initial trials when we tried out a few of the promising GNN models discussed above (GAT^35^, Graph Network^31^ and PNA^34^), we observed that contrary to our anticipation, they do not improve the damage prediction performance on our data set. This leads us to think that in our specific case, we might be restricted more by other factors. One candidate is the annotation noise that might be introduced during the annotation process due to the grain-level abstraction. But more dominant factors could be the lack of some critical information in the data and/or some essential information that is left hidden due to the simplified data representation.

A factor detrimental to all utilized models is the absence of three-dimensional information on the internal distribution of MnS inclusions in our experimental data. Moreover, subsurface microtexture information is limited to the electron interaction volume during the EBSD measurement and is thus minuscule. However, even irrespective of missing subsurface information, the graph, and conventional ML approaches did not consider all available contextual information. Pores and inclusions not being modeled explicitly as nodes in the structural graphs result in some spatial information loss during graph abstraction. Moreover, grain boundary properties such as the maximum disorientation $\Delta g_{max}$ or the maximum Young’s modulus difference across grain boundaries $\Delta C_{max}$ to *any* neighbor grain are considered as node features, but their spatial localization is discarded. Graph models, as opposed to conventional ones, can extract and incorporate this information to some extent from neighbor node features. Virtually all protrusions were observed not to cover the whole grain but rather be localized at specific grain boundaries, see Fig. 1a, b and c. This emphasizes the importance of spatial information and of the comprehensive description of individual grain boundaries. Especially in the case of irregular grain shapes, detailed morphological information of grain ensembles, e.g., acute grain boundaries, can be comparatively more relevant but is not provided to the graph models. The damage being localized to specific grain boundaries implies that not all neighbor grains are equally important to determine whether the target grain will contain a protrusion. The information on damage localization within a grain is experimentally available and can for instance be incorporated during training by assigning labels to edges, i.e. grain boundaries, rather than nodes or distinct graph representations.

The graph modeled in this study is homogeneous and composed of unlabeled and undirected edges, which is an under representation of the domain knowledge about fatigue damage formation. For instance, entities such as grain boundaries, triple points, pores, and inclusions, along with their properties, can be modeled explicitly within a heterogeneous structural graph to preserve spatial and structural information better. There are GNN variants that can handle this enriched information, such as heterogeneous deep graph convolutional network (HDGCN)^36^. In materials research, a heterogeneous representation of microstructure and application of heterogeneous GNN variants has been demonstrated for the prediction of continuum properties, including tensile strength and Young’s modulus^37^. Relational graph convolutional network (RGCN)^38^ is an extension of GCN, where a learnable parameter exists for each distinct relation type, thus making the node hidden state update dependent on the relation type. Extending on this, the structural graph can be further semantically augmented by infusing factual knowledge and thereby converting the data representation into a knowledge graph. Relations that could be modeled in this context are known connections and interactions between individual features, the influence of grain size on the local strengthening behavior, or even properties of individual grain boundary types known from bicrystal tests^39^. Knowledge graph embeddings (KGE) can then be learned using existing methods as surveyed by Gesese et alia^40^. Some of these methods take literal and knowledge incorporated in triples jointly into account. One successful example of the application of the KGE models is on scholarly data^41^, which utilizes these models to perform the task of author name disambiguation.

Few damage instances exist in the data set where slip markings propagated through a grain boundary, see Fig. 1b. Here, coupling between grains is evident and puts the separate treatment of grain instances in the tabular approach into question. The approach outlined here assumes that damage instances are independent of each other and can be inferred solely from the initial state of the local grain environment. The mutual interference between protrusion instances is deemed mostly negligible due to the HCF loading and the relevant specimen and microstructure scales.

To represent the full history of failure, it will be necessary to follow the physical damage process of damage accumulation, micro-crack initiation, microstructural and physical short crack growth, followed by long crack growth. The GNN-based initial damage prediction discussed here needs to be coupled with other graph ML-based modeling approaches to predict crack initiation and short crack growth. The fact that HCF cracks initiate even more infrequently than slip markings form, might necessitate utilizing physics-informed ML approaches or supplementing synthetic data from knowledge-based simulations to reduce the experimental training data demand. We argue that having distinct models for the different fatigue stages is important to account for their fundamentally different underlying mechanisms and statistics. The same potentially applies to protrusions that emerged at vastly different cycle numbers (primary and secondary protrusions) or at different defects (pores or grain boundaries). Such different damage instances can be distinguished efficiently by the data-processing workflow outlined in our previous work^21^ through the post-processing of the in-situ image series and the spatially correlated high-resolution pore information, respectively.

## Conclusions

Considering the complexity of predicting grain-wise cyclic damage accumulation, the prediction performance is very promising. The graph models were trained directly on the graph representations of experimental microstructures. They outperform standard machine learning models and the phenomenological crystal plasticity model substantially and also learn more nuanced relations. Interpretability techniques such as integrated gradients enable insights into which features are considered relevant and even permit the detailed assessment of individual interesting grain ensembles. Integrated gradient attribution reveals that graph convolutional networks learn a comparatively balanced representation taking into account all feature types (morphological, crystallographic, micromechanical, and grain boundary-related). The dependence plots give insights into how feature values affect damage prediction and indicate mechanistically plausible relations that are aligned with current fatigue literature. For instance, such analyses suggested that high values of the GB crack initiation metric^30^ also promote the mechanistically-distinct slip marking formation.

At the same time, the results presented here also indicate that the feature set might not be completely discriminative for the problem at hand, especially at the lower loading amplitudes. Indeed, information on subsurface microstructure and volume defects, known to be present in the material, are not captured in the features. Moreover, the feature quality and hence a meticulous microstructure reconstruction is crucial but might not be adequate for grains under lower loading conditions. Aside from feature completeness and quality, the data quantity is still a limiting factor. To tackle all aforementioned points, a multimodal X-ray diffraction contrast tomography and phase-contrast computed tomography data set is being acquired. Such a data set containing three-dimensional microtexture and volume defect information will then enable a thorough analysis of microstructural driving forces utilizing the combination of powerful graph-based models and appropriate interpretability techniques. Compared to the common statistical techniques, this approach could bring about a considerable improvement in analyzing microstructure-based predictions, potentially finding elusive patterns that are yet unknown to the fatigue community.

## Materials and methods

### Data set generation

The data from which the tabular and graph representations are derived was acquired by several measurement techniques. A detailed description of the employed characterization and its process parameters is outlined in Durmaz et al.^21^. This section presents a summary of the data extraction process.

The investigated material is high chromium alloyed EN 1.4003 ferritic steel. In terms of chromium content, it exceeds the solubility limit in iron. From etched longitudinal and cross-sections, manganese sulfide type II and III inclusions and finely distributed precipitates, as well as decorated grain boundaries, are observed. Presumably, Zener grain boundary pinning at aforementioned particles during grain growth causes irregular grain shapes, see Fig. 1. From a steel rod, mesoscale planar fatigue specimens were prepared through a series of fabrication techniques comprising wire discharge machining, laser cutting, and a series of metallographic grinding, electropolishing, and polishing steps with a colloidal silica surface finish. Prior to fatigue, the undisturbed microstructure of the fatigue specimens’ surfaces was investigated by electron backscatter diffraction (EBSD). Along those lines, a tile image series capturing the whole highly loaded surface with topography-sensitive imaging (Everhard–Thornley detector) in scanning electron microscopy (SEM) was acquired and stitched subsequently. This modality acted as an undeformed reference in which image distortions are largely absent and allowed for preserving high-resolution features while facilitating large scan areas. Analogously, but at slightly higher magnification, after fatigue, such a stitch image routine was repeated to capture local damage formation events. This data was then passed to a convolutional neural network trained to segment damage instances, including protrusion slip markings and cracks^2^ as classes to generate a semantic damage mask, followed by performing some manual corrections on it. Fatigue testing in the HCF regime was performed through bending resonant fatigue testing with mesoscale specimens. Ascribing to the testing concept and its pronounced sensitivity, early fatigue states can be characterized including the formation of surface slip markings. Such slip markings within grains occurred predominantly in the surroundings of grain boundaries as depicted in Fig. 1a,b and c.

The data utilized in this study originate from four specimens that were exposed to a distinct cyclic load corresponding to von Mises stress amplitudes ranging from 240 to 262 MPa with a load ratio of R =$\,-1$. All aforementioned preliminary microscopy techniques were performed on both sides of the planar specimens in order to increase the data quantity. Due to the scarcity of common image features of the highly polished specimen surfaces in the distinct modalities, multimodal registration necessitates the manual selection of point correspondences. Pores and residual surface contamination acted as characteristic features which were visible in the different modalities. A multi-stage registration procedure was followed where mostly linear similarity transformations were applied to the image data. The EBSD data posed an exception for which an additional elastic transformation was performed to correct for its inherent spatial distortion.

The multimodal data described above was represented as a graph $G = (V,E)$, where each EBSD-reconstructed grain is abstracted as a node $v \in V$. A set of features as described in ’Feature engineering” Section were computed for each grain ($x_v \forall \,v$) and are stacked vertically to build a feature matrix *X*. The adjacency information of grains from EBSD data was used to connect grain nodes with their neighbors through edges *E*. The edges are represented as an adjacency matrix *A*. This results in an undirected homogeneous graph, i.e., single node type and relation type. The microstructural graph data set, in the following referred to as a structural graph, could also be simplified into a tabular representation with only the grain features *X* as inputs and the corresponding damage labels as output. Such a representation lets us use conventional machine learning techniques which can be then compared with graph-based approaches. Grain-wise damage information was considered as ground truth for supervised training and is represented as a binary array *y*. Owing to minor residual misalignment after registration and the tendency of damage instances to be situated immediately at grain boundaries, the damage instances’ assignment to individual grains was corrected by visually inspecting the SEM image after fatigue. Whenever a part of a slip marking extended into a grain, it was considered damaged.

The complete data set derived from the multimodal data contains a total number of 7633 grains when discarding individual microtwins. Instead, the proportion of twin boundary length contributing to grain was considered as a feature. Owing to the HCF loading, only 311 grains among all grains contained surface slip markings. All kinds of protrusions are taken into account, irrespective of their time of emergence. This includes also protrusions that emerged at pores. Microstructurally short cracks and surrounding crack-induced plasticity are not considered in this task. Hence, the data set comprises a rather pronounced inherent imbalance where only 4.25% of the grains exhibit fatigue slip markings. An underlying assumption is that in the HCF regime and the grain sizes of the material at hand, the localized damage instances, and both specimen sides are mechanically decoupled from each other.

### Feature engineering

In order to predict the emergence of protrusions within grains by learning an ML classifier, a set of descriptive features needs to be computed. Since environmental, as well as surface topography influence factors, were largely suppressed in this study, and only one material was investigated in the context of fatigue, the focus was placed on capturing microstructural descriptors and mechanical loading comprehensively. This entails microstructure morphology, crystallography (microtexture), and pore defect attributes as well as hybrid features that couple mechanical load with microstructure. A list and rationale for the features are provided in Table 4 and “Discussion” Section, respectively. For instance, the grain size is an important feature as it affects the mean free path of dislocations in the absence of precipitates and, therefore, the local strengthening behavior. Further features are considered, which are slightly altered permutations and variants of the ones in Table 4. In total, 120 grain-level descriptors were considered to capture various aspects of the microstructure, pore defect distribution, and loading. However, some of these engineered features are highly correlated. In particular, this applies to the features addressing grain morphology. Since some features considered not only individual grains but also differences with respect to adjacent grains, some contextual information is fed in during model construction. All features were imputed, i.e., missing values were filled by applying the mean value. Subsequently, all features were standardized to remove the mean and scale to a variance of unity.

The set of features is extracted using an automated Matlab routine including different functionalities of the image processing and computer vision toolbox in combination with MTEX, a third-party toolbox providing a variety of crystallographic routines^45^. Before feeding these features to ML approaches, various feature selection or dimensionality reduction techniques could be beneficial. In our case, principal component analysis (PCA)^46^ was applied optionally. PCA is arguably the de facto standard for linear dimensionality reduction. The approach projects the features into a selected few eigenvectors of the data set (called principal components). The principal components are chosen based on the eigenvalues, ensuring that we select a smaller subset of directions that could capture most of the data sets’ variance.

### Graph-based machine learning approaches

In our graph data set, grains are represented through nodes where nodes of adjoining grains are connected by binary edges as described in “Data set generation” Section. Each grain node has a set of features computed for describing itself and sometimes its environment, as presented in “Feature engineering” Section. For the supervised training of ML algorithms, the damage annotation of each grain node is used. The graph data set presented is undirected and homogeneous.

**Figure 5:**
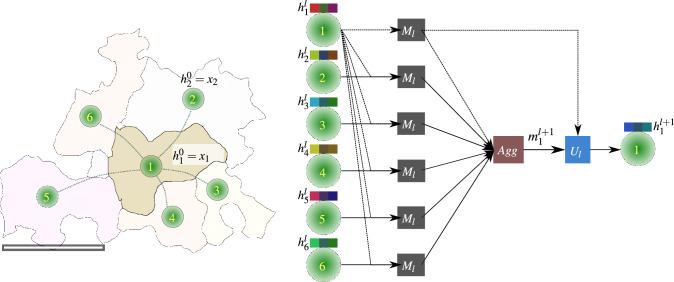
Figure illustrating how features are propagated in a single layer of GNN for a target node using the MPNN framework. The example here corresponds to the microstructure graph from Fig. 1d. The micron bar in the Fig. corresponds to 50 $\upmu$m.

Graph neural networks are a class of ML models designed for using data represented as graphs. In a nutshell, each layer of a GNN updates a node’s representation (called hidden state representation, $h_{v}^{l}$ after layer *l*) by considering both its previous state as well as information from its neighborhood. The initially hidden state representation of all nodes is the input features, $x_v$. There are numerous types of GNNs, which vary in how the so-called *message passing* and *update* phases are defined. Many of these GNN types could be generalized under a message-passing neural network (MPNN) framework, described in Gilmer et al.^10^. An MPNN layer describes the update of a target node representation within both phases. During the message passing phase (parameterized by the message function $M_l$), each node in the neighborhood of a target node *v*, $u \in N(v)$, and optionally the target node itself creates a message. These messages are derived from the previously hidden state representations of the corresponding nodes $h_{u}^{l} \forall u \in N(v)$ and $h_{v}^{l}$, and these messages are then aggregated in a permutation-equivariant way. Subsequently, in the update phase (defined by the update function $U_l$), the aggregated message ($m_{v}^{l+1}$) is used for updating the hidden state representation of the target node. After the two phases of an MPNN layer, each node in the graph is converted into an embedding that contains information about its neighborhood. When *N* such MPNN layers are applied, then each node acquires information from nodes that are *N* hops away. These embeddings are then used further to get the desired output. An example of a single MPNN layer updating the hidden state representation of the central node in Fig. 1b is illustrated in Fig. 5. For an undirected homogeneous graph, an MPNN update of the hidden state representation of a node *v* can be written as follows (note that we assume element-wise summation of messages as the aggregation operation):

1 $$\begin{aligned} m_{v}^{l+1}= & {} \sum _{u\in N\left( v\right) }M_l\left( h_{v}^{l}, h_{u}^{l}\right) \end{aligned}$$

2 $$\begin{aligned} h_{v}^{l+1}= & {} U_l\left( m_{v}^{l+1}, h_{v}^{l}\right) . \end{aligned}$$

**Table 5:** Graph convolutional network (GCN) and graph isomorphism network (GIN) defined as per MPNN framework described in 1 and 2.

Model	$M_l$	$U_l$
GCN	$c_{v}^{u}h^{l}_{u}$, where $c_{v}^{u}=(deg(v)deg(u))^{-\frac{1}{2}}A_{v,u}$	$ReLU(W^{l}m_{v}^{l+1})$
GIN	$h_{u}^{l}$	$MLP \left( \left( 1+\epsilon \right) h_{v}^{l} + m_{v}^{l+1} \right)$

In this study, we consider the GNN types GCN and GIN. They can be distinguished by their message and update functions, as shown in Table 5. In a GCN model, the message function is a fixed transformation that depends only on the graph connectivity, represented by the corresponding adjacency matrix element $A_{v,u}$, and node degree (*deg*), i.e., the number of edges connected to a node. It is to be noted that a message is also made by the target node itself. Also, each message only considers the hidden state representation of the node that’s generating it. The aggregated messages are then used to update the target node’s hidden representation using a trainable matrix $W^{l}$. It can be noted that this weight matrix is shared across all nodes in the same GCN layer. Meanwhile, in a GIN model, the update function uses a more expressive MLP to update the node features based on aggregated messages. However, it is to be noted that in GIN the messages themselves are just the previous hidden layer representations of the neighbor nodes. Both GCN and GIN have an inductive bias that treats features of the immediate neighbor nodes as more important than ones that are far apart.

We employed a transductive training setting in our experiments. In this setting, both training and validation data will be seen by the model during training but only the labels of the training data will be provided. The trained model will then try to predict labels of the validation data during evaluation. The grain features were carefully checked to prevent information leakage between the training and validation set.

### Interpretability of machine learning models

ML models can be understood and trusted better by the use of interpretability techniques that shed light on how the models make predictions. One of the very basic interpretability techniques is attribution which tells us how important particular parts of a specific input to an ML model are for predicting a particular output. Integrated gradients is one such attribution method and one of the recommended methods for graph neural networks^47^. IG is presented by the authors as a technique that satisfies several of the axioms that are considered desirable for an interpretability technique—completeness, linearity, symmetry preservation, sensitivity, and implementation invariance^27^.

IG attribution value is calculated for each input feature (pixel, voxel, text, value, etc.) with reference to a baseline. The baseline input is an input that represents an absence of an input signal provided to the model. Let $F:{\mathbb {R}}^{n}\rightarrow \left[ 0,1 \right]$ be the deep learning model and $x \in {\mathbb {R}}^{n}$ be a particular input to the model and $x' \in {\mathbb {R}}^{n}$ be the baseline input. Then IG can be calculated as the path integral of the gradients of *F* for the straight line path from $x'$ to *x*. In other words, we compute gradients at all points in the straight line connecting $x'$ and *x*, and then accumulate these gradients. The IG value for the $i$th dimension of an input *x* and baseline $x'$ is defined as follows:

3 $$\begin{aligned} IG_i(x) {:}{:}= (x_i - x_{i}^{'}) \times \int _{\alpha =0}^{1} \frac{\partial F(x'+\alpha \times (x-x'))}{\partial x_i} \mathrm {d\alpha } \end{aligned}$$

In our specific case, we computed IG attributions separately for nodes and edges. For calculating node attributions, we used a baseline that has the same topology as the input graph. So, the difference between baseline and input was only in the node features. The node features were interpolated for the segments between a baseline and the input value at hand and gradients accumulated. Similarly, for the edges, we used a baseline that differed only in the topology from the input graph. The baselines for nodes and edges were chosen to be values that represent a complete absence of signals. For nodes, this could be approximated by the mean feature value in the data set. In our case, since we passed standardized features to the models, the mean value of all features was zero. Hence, we could use a null graph as the baseline, i.e., a graph with all feature values as zero but the same topology. For the edges, a complete absence of signal could be approximated by a graph with no edges, and this was the baseline we used. We used Gauss-Legendre integration to find the path integral and tuned the number of steps based on the convergence delta.

These IG attribution values could then be aggregated per node or per feature to compute node-wise as well as feature-wise IG attribution values. Thus using the IG attribution values, one can look at how important each node, edge, and specific feature dimensions are for the GNN model. We use the IG attribution functions from the Captum library^48^.

Random forest models could also be made more interpretable by looking into the importance of different input features for the models. We used a metric called mean decrease in impurity (MDI)^49^, as implemented in scikit-learn^50^. The MDI score is calculated for each feature by taking a weighted average of the decrease in the impurity measure across all nodes in all the decision trees. The probability of reaching a node was considered as the weight while averaging. The impurity metric describes the homogeneity of the labels at a node in the decision tree, and during training of a random forest, a split is found for each node based on those features which decrease the impurity of that node the most. We used entropy as the impurity measure when training BRF models.

### Supplementary Information

Supplementary Information.

## Data Availability

The tabular and graph data sets generated and analyzed for this study can be found at http://dx.doi.org/10.24406/fordatis/248.

## Code availability

The codes used in this study are available from the authors upon reasonable request.
