# Author Correction: Materials fatigue prediction using graph neural networks on microstructure representations

**DOI:** 10.1038/s41598-023-40772-8

**Published:** 2023-08-21

**Authors:** Akhil Thomas, Ali Riza Durmaz, Mehwish Alam, Peter Gumbsch, Harald Sack, Chris Eberl

**Affiliations:** 1 Fraunhofer Institute for Mechanics of Materials, Freiburg, Germany; 2 Chair of Micro and Materials Mechanics, Department of Microsystems, University of Freiburg, Freiburg, Germany; 3 Télécom Paris, Institut Polytechnique de Paris, Paris, France; 4 Institute for Applied Materials-Reliability and Microstructure (IAM-ZM), Karlsruhe Institute of Technology, Karlsruhe, Germany; 5 FIZ Karlsruhe–Leibniz Institute for Information Infrastructure, Karlsruhe, Germany; 6 Institute for Applied Informatics and Formal Description Systems (AIFB), Karlsruhe Institute of Technology, Karlsruhe, Germany

Correction to: *Scientific Reports* 10.1038/s41598-023-39400-2, published online 02 August 2023

In the original version of this Article the figure legend of Figure 3 was incomplete, where the color codes used in panels **c**, **d** and **e** were not defined.

“Images showing interpretability analysis results of the GCN model from Table 1 using integrated gradients (IG) attribution (**a**)–(**d**) and the BRF model using the mean decrease in impurity (MDI) metric (**e**). Subfigure (**a**) shows a damaged target grain (#4641) predicted correctly by the GCN model. The microstructure graph and damage (white) are overlayed on the IPF-colored microstructure image. The more opaque the nodes and the edges are, the higher their IG attribution value and thus the higher their importance for the prediction outcome. To complement this, subfigure (**b**) shows the secondary electron SEM image of the damage instance. Subfigure (**c**) shows the importance of specific features for making the particular prediction in subfigure (**a**). In contrast, in (**d**) the joint importance aggregated over all true positive instances in the data set is displayed. The color code and feature symbols for the bar plots in subfigures (**c**)–(**e**) refers to the feature taxonomy introduced in Table 4. Similarly, the cross and check marks are transferred from Table 4 to indicate features that utilize information on adjacent grains.”

now reads:

“Images showing interpretability analysis results of the GCN model from Table 1 using integrated gradients (IG) attribution (**a**)–(**d**) and the BRF model using the mean decrease in impurity (MDI) metric (**e**). Subfigure (**a**) shows a damaged target grain (#4641) predicted correctly by the GCN model. The microstructure graph and damage (white) are overlayed on the IPF-colored microstructure image. The more opaque the nodes and the edges are, the higher their IG attribution value and thus the higher their importance for the prediction outcome. To complement this, subfigure (**b**) shows the secondary electron SEM image of the damage instance. Subfigure (**c**) shows the importance of specific features for making the particular prediction in subfigure (**a**). In contrast, in (**d**) the joint importance aggregated over all true positive instances in the data set is displayed. The color code and feature symbols for the bar plots in subfigures (**c**)–(**e**) refers to the feature taxonomy introduced in Table 4. The color code in (**c**)–(**e**) refers to the type of the feature: gold refers to “morphological and topological features”, cyan refers to “crystallographic orientation, misorientation and quality-related features”, and red refers to “micromechanical and loading-related features. Similarly, the cross and check marks are transferred from Table 4 to indicate features that utilize information on adjacent grains.”

The original Article has been corrected.

