# Supplementary material for: Materials fatigue prediction using graph neural networks on microstructure representations
Source: Sci Rep. 2023 Aug 2;13:12562. doi: 10.1038/s41598-023-39400-2 (PMC10397301; doi:10.1038/s41598-023-39400-2)
Supplement: Supplementary file 1 — Supplementary Information. [file 41598_2023_39400_MOESM1_ESM.pdf]

Materials fatigue prediction using graph neural networks on microstructure representations

Supplemental

Akhil Thomas, Ali Riza Durmaz, Mehwish Alam, Peter Gumbsch, Harald Sack, and Chris Eberl

Supplementary note 1: Model evaluation metrics

In the main publication, we use the metric depicted in Supplementary Table 1.

Supplementary Table 1: Different relevant evaluation metrics based on the confusion matrix elements, i.e., true positives (TP), false positives (FP), true negatives (TN) and false negatives (FN).

**Precision**	**Recall**	**F_1-score**	**Balanced Accuracy**
$$PPV = \frac{TP}{TP+FP} \quad (1)$$	$$REC = \frac{TP}{TP+FN} \quad (2)$$	$$F1 = 2 \cdot \frac{PPV \cdot REC}{PPV + REC} \quad (3)$$	$$BA = \frac{1}{2} \cdot \left(\frac{TP}{TP+FN} + \frac{TN}{TN+FP} \right) \quad (4)$$

Supplementary note 2: Additional information on features

For some features utilized in the main publication, a detailed definition is provided in Supplementary Equations 5–9. The kernel average misorientation assesses *local* disorientations within a $N \times N$ kernel and describes the average of local disorientations between the center pixel and each surrounding pixel contained in the kernel.

$$KAM_{i,j} = \frac{1}{N^2 - 1} \sum_{(k,l) \in N_N(i,j)} \omega(g_{i,j}, g_{k,l}) \tag{5}$$

Here $N_N(i, j)$ denotes the subset of neighbor pixels for the center pixel at position $(i,\ j)$ and $\omega(g_{i,j}, g_{k,l})$ the disorientation between the center pixel and another pixel within the kernel. Whenever the kernel extended into a neighbor grain, these pixels were not considered in the set of neighbor pixels $N_N(i, j)$. This pixel-wise metric was then averaged over all pixels contributing to the target grain to obtain $\overline{KAM}$.

A metric that characterizes *grain-level average* disorientations is represented by the grain orientation spread (GOS). This metric leverages the mean grain orientation $\overline{g_n}$ by averaging the disorientation at each pixel contained in the grain with respect to it $\omega(\overline{g_n}, g_p)$.

$$GOS_n = \frac{1}{N_p} \sum_{p=1}^{N_p} \omega(\overline{g_n}, g_p) \tag{6}$$

The subscripts n and p are indices for the grain and pixel, respectively. GOS_n directly represents the feature listed in the feature table of the main publication.

The slip transmission factor[1] provides a metric on how well slip systems across a grain boundary are aligned. Therefore, the angle between the slip directions κ and the slip plane normals ϕ is considered and their cosines are multiplied.

$$m' = \cos(\phi) \cdot \cos(\kappa) \tag{7}$$

This feature was computed either as the maximum and mean of all grain boundaries contributing to the target grain or at specific grain boundaries where the directional grain size in slip trace direction was largest. A uniaxial stress state was assumed to determine the activated slip planes.

Another feature assessed for grain boundaries is the so-called misorientation crack factor M_{ig} [2]. Similar to m', it relies on a slip system misorientation metric, in this case, in the rotation vector $\mathbf{\Delta R}$. It considers the cross product between the slip plane normal $\mathbf{n}$ and slip direction $\mathbf{s}$ per grain and then computes the element-wise difference between adjacent grains. The authors [2] argue that this $\mathbf{\Delta R}$ factor causes an opening or closing force at the grain boundary at the tensile half cycle. The rotation vector $\mathbf{\Delta R}$ is then projected onto the grain boundary trace unit vector $\mathbf{e_y}$ resulting in an incompatibility factor. Since normal forces on the grain boundary also cause an opening, the ratio between the normal stress component $\sigma_{N,GB}$ to the externally applied uniaxial stress σ_0 is also considered.

$$M = \frac{\sigma_{N,GB}}{\sigma_0} \cdot \mathbf{\Delta R} \cdot \mathbf{e_y} \quad \text{with} \tag{8}$$

$$\mathbf{\Delta R} = (\mathbf{n} \times \mathbf{s})_{G2} - (\mathbf{n} \times \mathbf{s})_{G1} \tag{9}$$

This metric M applies to an individual grain boundary. The M_{ig} feature which was utilized for damage prediction

was computed as the maximum and mean M of all grain boundaries contributing to the target grain. A uniaxial stress state was assumed to determine the activated slip planes.

It can be noticed that features like EBSD confidence index and image quality are included in the feature set. These features contain information about contamination of the specimen surface as well as polishing quality. They act as quality features providing information about the state of the specimen surface, which is not available elsewhere. Additionally, EBSD confidence index could be even giving us clue to dislocations.

Supplementary note 3: Baseline model

As a baseline for the data-centered models presented here, a rule-based crystal plasticity finite element model for a fatigue load path was utilized. This simulation routine was applied directly to one of the measured microstructures retrieved by EBSD. The simulation routine was adopted from [3]. Since there the constitutive modeling is outlined in detail, in the following, only the most important underlying model assumptions are presented. Aside from this, the specific data processing which transcribes the full-field stress and strain information into a grain-wise binary damage indicator for the baseline is described.

The constitutive model assumed

- plasticity through dislocation slip on $\{1\,1\,0\}\langle 1\,1\,1\rangle$ as the sole mechanism of plastic deformation,
- Schmid behavior, i.e., only shear components on permitted slip systems contributing to plastic deformation,
- a local model which does not explicitly model long-range interactions or grain size effects, i.e., the critical resolved shear stress (CRSS) which needs to be exceeded to trigger dislocation movement solely depends on the deformation state of the individual element [4],
- small strains,
- prismatic grain structure,
- absence of intragranular misorientations, i.e. the mean orientation was applied throughout the grain, and
- different so-called fatigue indicator parameters (FIP) containing information on damage formation on the microscopic scale, i.e. for individual grains.

The FIPs translate the mechanical fields into a damage accommodation tendency. Applied FIP comprised Fatemi-Socie [5, 6], accumulated plastic slip [7, 8], and dissipated energy [9]. Since the FIP values are continuous and can exhibit distinct ranges all metrics were normalized. Further, the FIPs were aggregated within each grain by considering their maximum values for the relevant set of pixels. In order to obtain a binary classifier from these continuous FIPs, a decision boundary was defined such that the maximum F1 score is attained on the ground truth grain level annotation.

Supplementary note 4: Training notes

Supplementary Table 2: Imbalance correction and hyperparameters for each of the ML model type.

Model	Imbalance correction	Hyperparameters
BRF	Balanced random forest	Number of trees, depth of trees, impurity metric
SVM	C parameter of RBF set using class weights	Parameter C, beta of RBF kernel
MLP	Weighted cross entropy loss	Learning rate, number of MLP layers, hidden units in layers, learning rate
GNN	Weighted cross entropy loss	Layer type, learning rate, number of GNN layers, number of pre-/post-MLP layers, batch norm, hidden units in layers

The graph representation of the data is stored as a pytorch-geometric data set ([10]) and the tabular representation is stored as HDF5 file for easy processing. Both data sets uses the same index of grains. Then the data is split into train and test sets using a stratified five-fold sampling to ensure the same distribution of damage instances in each fold despite their scarcity.

Balanced Random Forest

A balanced random forest implementation from the imbalanced learn ([11]) python library was selected. A random search in a defined parameter space allowed identifying a presumably near-optimal set of hyperparameters. The number of individual trees contributing to the model was set to 1000, their maximum depth was kept unrestricted. Each decision tree was trained with a bootstrap sample of seven randomly selected features and we used entropy as the impurity metric. Leaf nodes with a minimum sample of one were permitted and post-pruning was omitted.

Support Vector Machine

A support vector machine implementation from scikit-learn ([12]) python library was used for the experiments. A radial basis function (RBF) kernel was selected and its parameters C and gamma were optimized using random search, finding a value of 3.25 and 9.40e-18 respectively. The parameter C was then adjusted for each class separately inversely proportional to the class frequencies of the data set.

Multilayer Percepton

The multi-layer perceptron (MLP) implementation from scikit-learn ([12]) python library was used. For hyperparameter optimization, the ray tune ([13]) python library was used. The near-optimal MLP we found had 5 fully connected layers with each having a hidden layer size of 128. The trainings used weighted cross entropy loss using inverse class frequencies of the data set as the weights. Adam optimizer with a base learning rate of 1e-4 and a weight decay of 5e-4 was used. Drop-out regularization [14] with a factor of 0.8 was applied.

Graph training

The implementation of graph convolutional and graph isomorphism networks (GCN, GIN) from pytorch-geometric ([10]) python library was used. GraphGym [15] python library was used for performing hyperparameter optimizations. Both models used 2 layers of the respective message passing layer followed by 2 layers of MLP, with all layers having 64 hidden units. Adam optimizer wth a base learning rate of 8e-3 and weight decay of 5e-4 was used in the training. Batch normalization [16] as well as drop-out regularization [14] with a factor of 0.2 was applied. Trainings by default used weighted cross entropy loss similar to MLP training.

Supplementary note 5: Performance of the balanced random forest model

Supplementary Table 3: The confusion matrix of the balanced random forest classifier for the binary damage classification.

		Prediction	
		Negative	Positive
Actual	Negative	5315 ★	2007 ★
	Positive	67 ■	244 ■

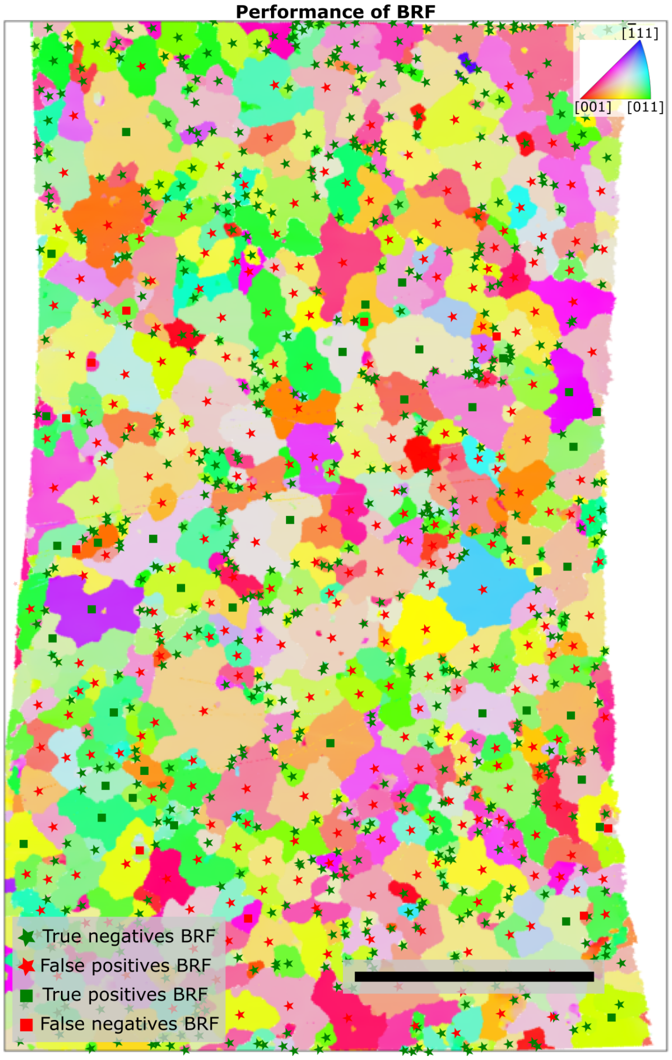

Supplementary Figure 1: Prediction performance of balanced random forest (BRF) model visualized by overlaying on an inverse pole figure color-coded microstructure map of P2_8 specimen marked side. Here, at each grain's centroid, a symbol is plotted that classifies the model's prediction in terms of confusion matrix elements. The "square" and "star" symbols indicate damaged and undamaged grains, respectively. A green colored symbol indicates that the model's prediction for that grain is correct (i.e., either true positive or true negative depending on the symbol), and a red symbol indicates a wrong prediction (i.e., false positives and negatives). The reference direction of the inverse pole figure (IPF) is the specimen normal (ND) [0 0 1]. The micron bar corresponds to 200 µm.

Supplementary note 6: Results table with precision and recall values

Each of the machine learning models were trained on five folds and their results on validation sets of the five folds are concatenated to get the presented results. The performance of the best models from each ML type is presented in supplementary Table 4. This is an extension of the table from main text with precision and recall scores as well as for the best models. The standard deviation of different ML models on the five-folds are also listed in the table.

Supplementary Table 4: Performance of machine learning models predicting the formation of protrusions. The table compares machine learning approaches operating on the tabular representation of data (balance random forests, support vector machines, and multilayer perceptrons) with those using graph representation directly (graph convolutional network and graph isomorphism network). The three columns on the left evaluate the models on the whole data set (aggregating across validation sets of all five folds), the fourth column shows the standard deviation across the five folds, and the rest of the columns evaluate them only on grains from a single specimen side for which a CPFEM phenomenological model predicting Fatemi-Socie fatigue indicator parameter is available as a baseline. Some special cases of models were also presented — the GCN and BRF models trained using data transformed by principal component analysis (PCA) and the GCN model trained with binary cross entropy loss without any additional imbalance correction techniques.

	**Complete data set**				**P2_8 marked**		
**Model**	**Precision**	**Recall**	**F_1-score**	$\sigma_{F_1\,(folds)}$	**Precision**	**Recall**	**F_1-score**
CPFEM Fatemie-Socie		—			0.19	0.35	0.25
BRF	0.11	0.79	0.19	0.01	0.12	0.78	0.20
BRF-PCA	0.11	0.78	0.19	0.01	0.12	0.86	0.22
SVM	0.22	0.29	0.25	0.04	0.22	0.29	0.25
MLP	0.21	0.45	0.29	0.02	0.24	0.53	0.33
GCN	0.25	0.50	**0.34**	0.02	0.31	0.61	0.41
GCN-BCE	0.31	0.32	0.32	0.03	0.38	0.45	0.41
GCN-PCA	0.27	0.47	**0.34**	0.02	0.34	0.62	**0.44**
GIN	0.28	0.41	0.33	0.02	0.36	0.51	0.42

Supplementary note 7: Study on fluctuations in prediction performance between the four specimens

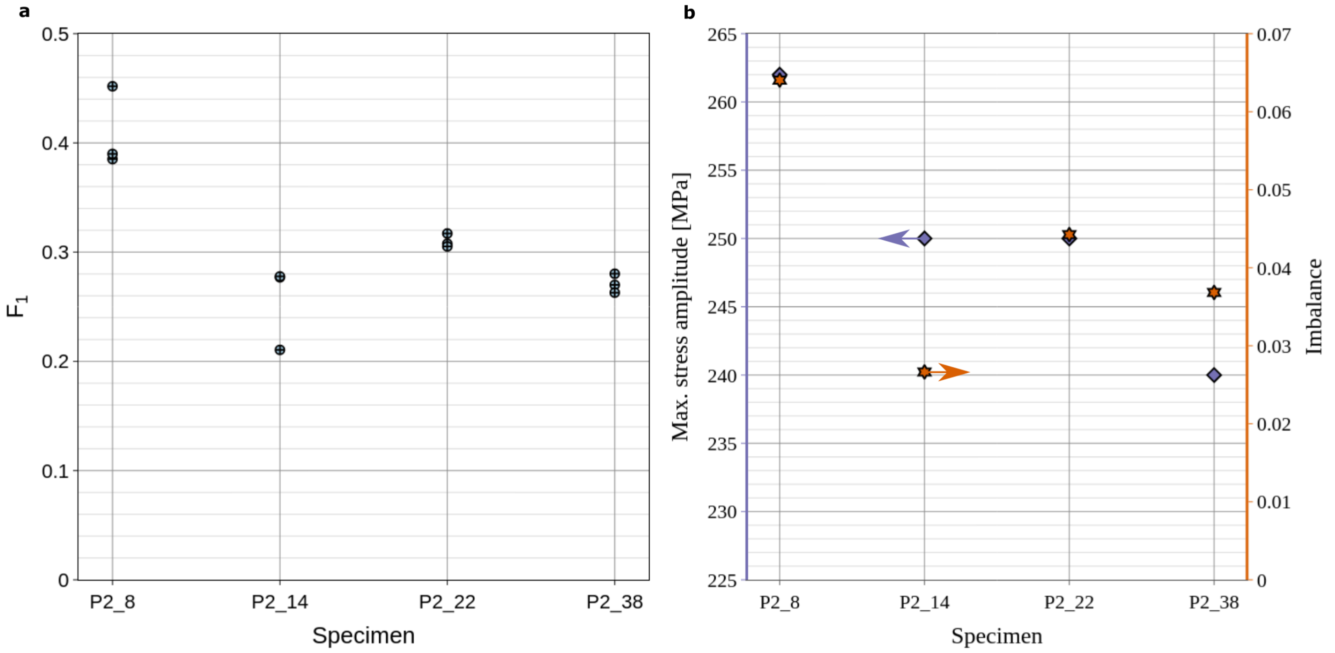

Supplementary Figure 2: Subfigure (**a**) shows the F_1-score of the GCN-PCA model from Table 4, evaluated per specimen. The results are computed by aggregating across the five folds. The model's performance for three different random initializations is shown in the figure. In subfigure (**b**), the maximum von Mises stress amplitude and imbalance ratio per specimen are plotted to unravel the reasons for the trend observed in (**a**).

The graph models perform differently well on the distinct specimen and excel on the highest loaded specimen P2_8. It can be hypothesized that the remaining three specimens, owing to the lower applied cyclic stress, exhibit different damage mechanisms relying more on the presence of nearby pores/inclusions or other microscopic flaws. Information on such microscopic flaws is more difficult to capture and preserve during microtexture measurements and data processing, respectively. Hence, for the specimens exposed to a lower load, the feature set potentially does contain less relevant information to predict slip formation. On the other hand, at slightly higher stresses, elastic and plastic interactions might dictate the damage formation. To test this hypothesis, a small study is performed and is shown in Supplementary Figure 2.Here, the correlation between the maximum applied stress amplitude, the imbalance factor (the portion of damaged grains), and the GCN-PCA model's F_1-score is assessed specimen-wise. A correlation between maximum stress amplitude and F_1-score is observed, cf. Figure 2a and 2b, which underpins a mechanistic difference between the different specimens. The number of resulting damage instances in a specimen depends on the applied stress amplitude where the statistical probability of damage formation drops exponentially with decreasing stress amplitude. The fluctuating proportion of damaged grains (imbalance) per specimen, see Figure 2b, can affect the training statistically. Specifically, the learned decision boundary can be dominated by P2_8 damage instances if they outweigh the number of damage instances on the other specimen. This would have implications on how to ideally acquire and compose fatigue data for mining fatigue driving forces.

While the strong correlations demonstrated there does not prove causality, we consider it highly plausible that there is a superposition of two effects — a mechanistic change in damage formation between the specimens (1) as well as a statistical influence during learning owed to the fact that P2_8 contributes the most damage instances (2).

Supplementary note 8: Comment on transductive and inductive training

GNN models can be trained in a transductive or inductive setting. We opted for a transductive training setting due to the small data set size and the possibly evasive statistical patterns we aimed to find. Aside from low data quantity, there is significant variance in von Mises stress amplitude during fatigue testing (240–262 MPa) with seemingly different underlying damage mechanisms. This renders inductive training infeasible. Inductive training is effective when training data is statistically relevant and contains patterns that can help discern the target labels. In cases where such a condition is not fulfilled, it will be beneficial to train in a transductive setting where the patterns (not labels) present in the validation data could also be used by models during training. Provided more data, in the future, an inductive setting, where the model will not see both the validation data and labels during training, can be employed. The inductive approach would enable us to develop models that can generalize better to unseen data.

Supplementary note 9: Study on data set size

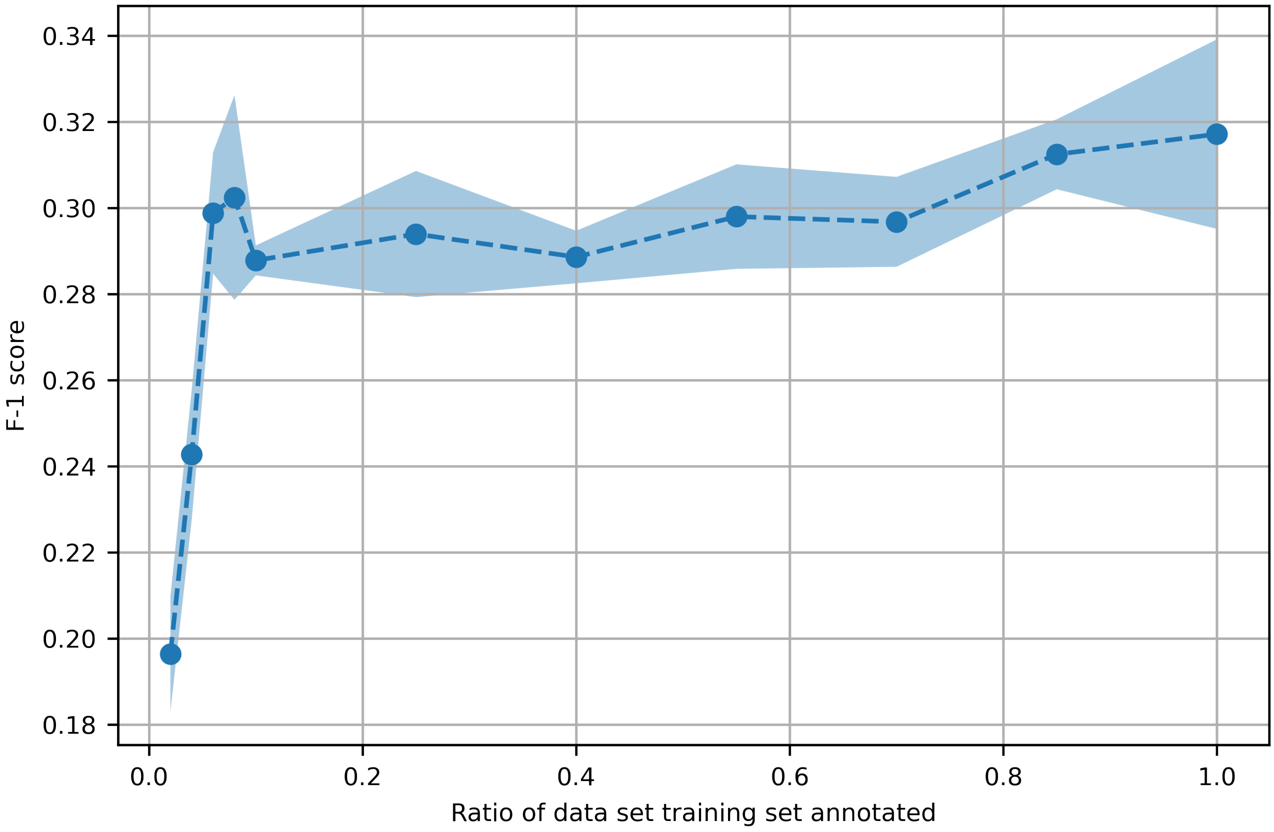

Supplementary Figure 3: Figure showing the influence of data set size on GCN model performance. Each point shows the mean and scatter in performance of the model when a fraction of the data set (x-coordinate) is provided with annotations during training. All data set fractions are tested against the same data set test set, which was not included in any of the trainings.

The performance of graph models relies on the quality and quantity of the data it was provided with. A study was conducted to see the influence of annotated data set size on the performance of GCNs. The GCN models were trained in a semi-supervised manner with only a fraction of the input data set provided with the annotation while training. All models were tested against the same test subset, which was not included in any of the trainings.

The results of the study can be seen in Figure 3. Here, we can observe that F_1-score of the models improve with more data given with annotations. Interstingly, an early jump in performance can be seen upto data set fraction of 0.1%. After that point, the performance still increases but at a relatively smaller rate. How the model would behave if we increase data quantity can be approximated by extrapolating the figure, which looks likely to saturate at a F1_score less than 0.6.

Supplemental References

1. Luster, J. & Morris, M. Compatibility of deformation in two-phase Ti-Al alloys: Dependence on microstructure and orientation relationships. *Metallurgical and Materials Transactions A* **26,** 1745–1756 (1995).
2. Blochwitz, C., Richter, R., Tirschler, W. & Obrtlik, K. The effect of local textures on microcrack propagation in fatigued fcc metals. *Materials Science and Engineering: A* **234,** 563–566 (1997).
3. Durmaz, A. R. *et al.* Micromechanical fatigue experiments for validation of microstructure-sensitive fatigue simulation models. *International Journal of Fatigue* **160,** 106824 (2022).
4. Sedaghat, O. & Abdolvand, H. A non-local crystal plasticity constitutive model for hexagonal close-packed polycrystals. *International Journal of Plasticity* **136,** 102883 (2021).
5. Fatemi, A. & Socie, D. F. A critical plane approach to multiaxial fatigue damage including out-of-phase loading. *Fatigue & Fracture of Engineering Materials & Structures* **11,** 149–165 (1988).
6. Przybyla, C., Prasannavenkatesan, R., Salajegheh, N. & McDowell, D. L. Microstructure-sensitive modeling of high cycle fatigue. *International Journal of Fatigue* **32,** 512–525 (2010).
7. Manonukul, A. & Dunne, F. P. E. *High- and low-cycle fatigue crack initiation using polycrystal plasticity* in *Proceedings of the Royal Society of London A: Mathematical, Physical and Engineering Sciences* **460** (2004), 1881–1903.
8. McDowell, D. L. & Dunne, F. P. E. Microstructure-sensitive computational modeling of fatigue crack formation. *International Journal of Fatigue* **32,** 1521–1542 (2010).
9. Korsunsky, A. M., Dini, D., Dunne, F. P. E. & Walsh, M. J. Comparative assessment of dissipated energy and other fatigue criteria. *International Journal of Fatigue* **29,** 1990–1995 (2007).
10. Fey, M. & Lenssen, J. E. Fast graph representation learning with PyTorch Geometric. *arXiv preprint arXiv:1903.02428* (2019).
11. Lemaître, G., Nogueira, F. & Aridas, C. K. Imbalanced-learn: A Python Toolbox to Tackle the Curse of Imbalanced Datasets in Machine Learning. *Journal of Machine Learning Research* **18,** 1–5. `http://jmlr.org/papers/v18/16-365.html` (2017).
12. Pedregosa, F. *et al.* Scikit-learn: Machine Learning in Python. *Journal of Machine Learning Research* **12,** 2825–2830 (2011).
13. You, J., Ying, R. & Leskovec, J. *Design Space for Graph Neural Networks* in *NeurIPS* (2020).
14. Srivastava, N., Hinton, G., Krizhevsky, A., Sutskever, I. & Salakhutdinov, R. Dropout: a simple way to prevent neural networks from overfitting. *The journal of machine learning research* **15,** 1929–1958 (2014).
15. Liaw, R. *et al.* Tune: A Research Platform for Distributed Model Selection and Training. *arXiv preprint arXiv:1807.05118* (2018).
16. Ioffe, S. & Szegedy, C. *Batch normalization: Accelerating deep network training by reducing internal covariate shift* in *International conference on machine learning* (2015), 448–456.
